# Explaining the visible and the invisible: Public knowledge of genetics, ancestry, physical appearance and race in Colombia

**DOI:** 10.1177/0306312715621182

**Published:** 2015-12

**Authors:** Ernesto Schwartz-Marín, Peter Wade

**Affiliations:** Department of Anthropology, Durham University, Durham, UK; Social Anthropology, School of Social Sciences, University of Manchester, Manchester, UK

**Keywords:** ancestry, Colombia, DNA tests, lay knowledge, physical appearance, race

## Abstract

Using data from focus groups conducted in Colombia, we explore how educated lay audiences faced with scenarios about ancestry and genetics draw on widespread and dominant notions of nation, race and belonging in Colombia to ascribe ancestry to collectivities and to themselves as individuals. People from a life sciences background tend to deploy idioms of race and genetics more readily than people from a humanities and race-critical background. When they considered individuals, people tempered or domesticated the more mechanistic explanations about racialized physical appearance, ancestry and genetics that were apparent at the collective level. Ideas of the latency and manifestation of invisible traits were an aspect of this domestication. People ceded ultimate authority to genetic science, but deployed it to work alongside what they already knew. Notions of genetic essentialism co-exist with the strategic use of genetic ancestry in ways that both fix and unfix race. Our data indicate the importance of attending to the different epistemological stances through which people define authoritative knowledge and to the importance of distinguishing the scale of resolution at which the question of diversity is being posed.

## Introduction

This article presents results of research conducted in Bogotá and Medellín, Colombia, that aimed to assess, mainly in focus group (FG) contexts, how different people engage with the knowledge generated by recent genomic research on ancestry and population diversity in Colombia when they ponder questions of human diversity, national identity, race, personal constitution and heredity. These people were located at diverse distances from the specialist field of population genetics – they included humanities students, Afro-Colombian activists, life sciences (LS) students, forensic technicians and criminal investigators.^1^

Our article makes two central arguments, both of which highlight how publics engage with genetic knowledge about human diversity in very uneven ways. First, in terms of the weight attributed to genetics in shaping individual and collective identities and behaviour, and especially in terms of the status accorded the concept of race, there was a marked divergence between those who came from a humanities background or were race-critical Afro-Colombian activists and all the others in the FGs, who were varied but generally had LS training or were involved with genetics in their work. Even though both sets of people agreed on some fundamental ideas about the Colombian nation and its diversity, they used genetic and racial idioms to markedly different degrees. This may seem predictable, but the current literature does not generally highlight the need to nuance ideas about how genetics is or is not shaping ‘public’ perceptions, according to the epistemic viewpoints of different segments of the public (but see Jacob, 2012).

Second, among those more disposed to accord genetics a high degree of authority and weight – who are the main focus of this article – there was a significant difference depending on whether they were thinking on a collective level, about Colombia as a nation seen in terms of a racialized regional diversity, or on an individual level, in terms of personal identities and constitutions. At the individual scale of resolution, there was a good deal of flexibility: many other factors shaped the person, and genes might be expressed or remain latent. This suggests that, in assessing patterns of ‘geneticization’ in society, we need to be attentive to the specific domains in which people are imagining and plotting relations of being and belonging.

## Race, genetics and heredity

The literature on the relations between genetic ancestry data and social identities has often been concerned with whether recent genomic science fosters the genetic reification of belonging and identities of ethnicity, race and nation (Brodwin, 2002; Nordgren, 2010; Nordgren and Juengst, 2009). An initial difficulty with the question posed in simple terms is that within the practice of genomic science itself there are varied tendencies. Some geneticists contend that common-sense categories of race – based on a broad division of humans into continental groups – have some genetic validity; others disagree (Bliss, 2012). Even the latter may tacitly reproduce race-like categories in their work, if they refer to the continental structuring of biogeographical genetic ancestry and use ancestry informative makers, which often refer to continental population categories (Fujimura and Rajagopalan, 2011; Fullwiley, 2008; Nash, 2015; Shim et al., 2014). Many geneticists, especially, but not only, in the United States, believe that race is a necessary category for measuring health disparities in the pursuit of social justice, and thus use the category in their work (Bliss, 2012; Smart et al., 2008). Most population geneticists thus reproduce, explicitly or implicitly, racial or race-like categories in their research, as these enter into ways of sampling, organizing and interpreting data; in the process they lend genetic weight to social categories of race (M’charek, 2005; Montoya, 2011). But genomic data both fix and unfix race: they can figure race-as-ancestry in multiple and probabilistic ways, and may detach phenotype from genotype; on the other hand, they can also appear to lend genetic weight to simple, familiar racial categories and genealogical belongings (Abu El-Haj, 2012). In short, genomic data present ‘an unlimited finity of differences and belongings, breaking down simple racial categories, while remaining structured by key ancestral reference points that are race-like in their evocation’ (Wade, 2014: 237).

In addition, social categories of race, ethnicity and nation have long circulated among different specialist scientific fields – population genetics, forensics, medicine and so on – and between specialist scientific and non-scientific domains – government, media, popular culture and so on. In the process, race both acquires and sheds genetic and other biological meanings. The specialist scientific sphere is not divorced from other less specialist domains, because: genetic information circulates into those domains, via a self-consciously public science; social categories are used to organize sampling and data; and geneticists and other people are interested in explaining the same kinds of problems – albeit in different ways – about health, belonging, history, roots, appearance, heredity and diversity (Jasanoff, 2004; Latour, 1993). Thus, any impact genomic research has on categories such as race, ethnicity and nation is subject to complex mediations.

As a result, the relation between genetic ancestry data and social identities is an ambivalent, ambiguous and multivalent matter (Schwartz-Marín, 2015; Wailoo et al., 2012). Genetic data can ‘remediate’ cultural and archival memory for British publics interested in Viking roots, rather than simply re-casting it in genetic idiom (Scully et al., 2013). Likewise, for US and British subjects seeking African ancestry, DNA testing forms part of a process of ‘affiliative self-fashioning’, in which many other elements play a part (Nelson, 2008). Genetic ancestry data produces varied reactions among Brazilian recipients and does not necessarily make much difference to their racial self-identifications (Santos et al., 2009). Work on US ideas about race and genetics shows that people engage with genetic science and race in nuanced ways, rather than in terms of straightforward determinism (Condit et al., 2004). A study of how DNA figures in diverse medical, forensic and archaeological practices in Europe indicates that traces of race can be materialized in subtle ways, and race is not simply made present as a stark genetic object (M’charek, 2013).

Our research builds on these studies, but pays special attention to how engagement with genomic data varies according to people’s epistemic positioning on the spectrum of knowledge production, and also according to the scale of the problem of diversity they are pondering. We explore audiences that, while many had some knowledge of genetics in general, did not generally have a previous interest in genetic ancestry and were not members of associations for patients with genetic disorders or of ethnic groups with a stake in tracing ancestry (Wailoo et al., 2012). While some of our FGs included a few of the roots-seekers who have been examined in the literature (along with the consumers of the webpages of genetic testing companies) (Nelson, 2008; Schramm, 2012; Tyler, 2008), most participants had not pursued projects of ‘applied genetic history’ (Sommer, 2012). Within this broad category of people with little vested interest in tracing genetic ancestry, we distinguish among people with different epistemological stances about authoritative knowledge. We also show how unevenly genetic data enter into lay people’s interpretations of race and ancestry. In many ways, such data reinforce existing ideas, among some sectors of the public, but a good deal depends on the level of resolution at which people are trying to explain things – the regional/collective or the individual/personal level.

We present data from a variety of FG participants – mostly students and thus well-educated and relatively young – and indicate the divergence between humanities students and Afro-Colombian activists, on the one hand, and people with an LS training, on the other. But we have chosen in this article to focus in particular on the latter category, which includes university students of the natural sciences and medicine in Colombia and some forensic technicians and criminal investigators, who had more direct experience with genetics – although not usually with genetic ancestry testing. Our interest in this group of people derives from their location in the ‘certainty trough’ (Mackenzie, 1999), in which those who are close to scientific knowledge, but as users rather than specialist producers, tend to treat it as certain, while those who actually produce the knowledge (here the genomic scientists) and those who are distant from it (here the humanities students and Afro-Colombian activists) are more disposed see it as uncertain. The reactions of the group located in the certainty trough suggests that, while they tended to feel quite comfortable talking about race, genetics, ancestry and diversity in the same breath, even they adopted different registers depending on the scale of resolution of diversity that was at issue.

## Methods

The ideas presented here are the product of 45 FGs or group interviews done in the cities of Medellín and Bogotá in 2011–2012.^2^ All of the participants have been anonymized. The FGs drew participants from four constituencies: university students, forensic technicians, volunteers in a genomics research project and Afro-Colombian activists (themselves university students). As noted above, we draw a broad contrast between ‘life sciences’ people and the ‘humanities and social sciences’ people. We label these broad groups ‘LS people’ and ‘HS people’, respectively. It may be objected that the composition of the FGs pre-determined to some degree this interpretive contrast, as dynamics within FGs can lead people who share some attitudes to emphasize consensus. Against this, we note that (a) the LS and HS sets were heterogeneous. Each set comprised two or three sub-categories of people; and (b) these two sets concurred on some things and differed on others: there was not a binary contrast on all issues.

The LS people comprised the following sub-sets:

1. University students of medicine and biological sciences, selected from five universities in Bogotá.
2. *Forensics*. These participants included users and producers of forensic genetics at the Instituto Nacional de Medicina Legal y Ciencias Forenses (National Institute of Legal Medicine and Forensic Sciences), the Fiscalía Nacional (Public Prosecutor’s Office) – laboratory technicians and attorneys – and members of the national police force’s School of Criminal Investigation.
3. *LATINA*. The respondents in this group were participants in an international project that was collecting anthropometric measurements and genetic ancestry data on samples of Latin Americans and complementing these data with participants’ self-perceptions of ancestry. In Colombia, this project was being carried out at a public university in Medellín. Most of these participants were university students, and most of them in the LS, but this group was more diverse, including some with no specialized LS background, for example high-school students, an amateur genealogist and even a self-declared White supremacist. Most participants had attended an introductory LATINA lecture on population genetics.

The HS people comprised these sub-sets:

1. University students in anthropology, literature, history and pedagogy from one university in Bogotá.
2. *Afro-Colombian activists*. Members of Afro-Colombian social movements, based in Bogotá, with a diverse range of political interests and agendas linked to the better recognition and socio-political inclusion of Afro-descendants in Colombian society.
3. A few of the LATINA participants, as noted above.

Participants whom we cite are identified by their FG, using the letters ‘FG’, followed by the word Forensics, LATINA, Afro-Colombian, or University. The University category is divided into LS groups (lettered A to E) and the humanities groups (lettered H-1 and H-2). The individual groups are presented in a list at the end of the article.

**Figure 1.**
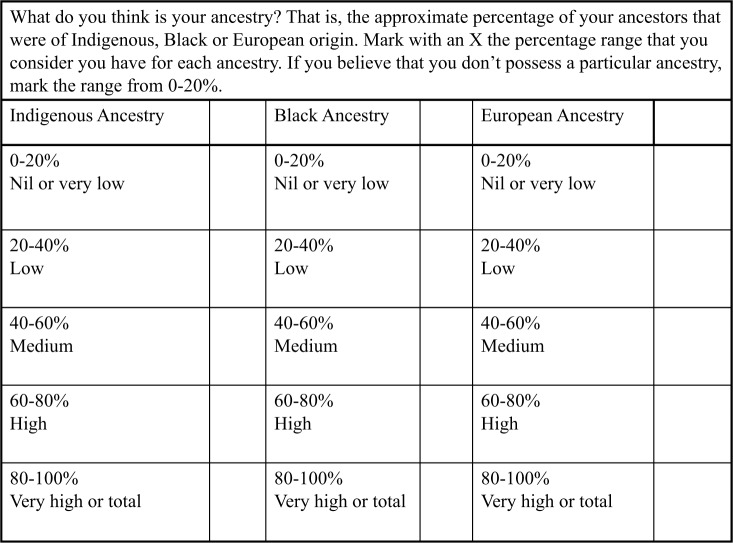
Ancestry table used in the focus groups.

Our FG sessions were divided into three segments. The first section used words and spontaneous associations to ascertain how people conceived of key notions such as *mestizaje* (roughly, racial and cultural mixture), nation, genetics and Colombia. We usually started with *mestizaje*, as this is a foundational concept for many Latin American ideas of nationhood, evoking the narrative that the nation was based on the biological and cultural mixing of European colonizers, indigenous Americans and African slaves; other notions then followed spontaneously. The second segment elicited participants’ ideas regarding genetic, racial and national difference through images that had been selected from accessible arenas of mainstream Colombian society, such as TV publicity about the national census (which in 2005 included a question designed to identify Afro-Colombians and indigenous peoples), and antiracist and human rights campaigns available on the Internet and on many billboards around Bogotá and Medellín. Finally, the activity ended with a mock genetic ancestry questionnaire (Figure 1). In this segment, we also asked participants to imagine that their DNA ancestry tests yielded results that were very different from their own estimates and probed them on whether they considered this outcome to be plausible and to explain why. Our aim was to assess how much authority people were willing to give to genetic data, even when these flew in the face of common sense. By forcing participants to speculate on a counter-intuitive scenario, we pushed them to challenge genetic determinism. The ancestry questionnaire was borrowed from its original context – the LATINA project – to explore its varied interpretations outside its original purpose. This was something new for all FG participants, except for those involved in LATINA, who had already filled the table before.

It is notable that the table used disparate categories: indigenous (*indígena*), Black (*negro*) and European (*europeo*). This was mainly because LATINA researchers believed that people would tend to avoid identifying ‘African’ ancestors, resulting in an underestimation of people’s perceptions of their Black African ancestry. The Spanish neologism *ancestría* (ancestry) was used in the table, a word which was unknown for most respondents, hence the explanation given in the table in terms of ‘your ancestors’. However, participants generally inserted the concept into their vocabulary, readily linking it with the more common concept of *ascendencia* (lineage, ancestry).

## The common sense of race, region, nation and genetics

In Colombia, race and region are closely linked, so that specific regions have powerful racial connotations (Schwartz-Marín et al., 2015; Wade, 1993). For example, the Pacific coastal region is seen as very Black (*negro*), while major areas of the Andean highlands are seen as predominantly mestizo and White (*blanco*). Ideas about racialized appearance and ancestry are often articulated in the idiom of regional difference. However, the word and concept of ‘race’ are not straightforward. Generally, we found that, when asked directly about race, participants in all the FGs spontaneously linked the term race to notions of purity and thought that in this sense, ‘race’ deserved to be treated with care in a country in which no one could be considered pure: ‘*todos estamos mezclados con todos*’ (everyone is mixed with everyone else; FG-University A). Many participants decided that since admixture was so generalized, races, in the sense of pure types, did not exist anymore. Others, including a few LS students, more radically defended the idea that races had never existed, citing the Human Genome Project’s finding that all humans are genetically very similar. Race was also debunked when humans were compared to other animal species: ‘very visible physical differences make it possible to talk about different races of dogs, but not persons’ (FG-University D).

HS people strongly resisted using the word ‘race’ and had real difficulties talking about the biological dimensions of heredity in the context of race and/or genetics. These participants commonly depicted race as ‘a social construction’, which sorted phenotypic diversity into arbitrary categories, used since colonial times to divide and oppress indigenous and Black people in Colombia. Their background in the humanities made linking race to genetics appear to them a politically retrograde and suspect move. As one humanities student said: ‘when genetics participates in the making of racial categories, it is participating in a discourse that is not naïve at all’ (FG-University H-1). Above all, all FG participants – except the White supremacist – were united in the view that *racism* was wrong.

Yet many FG participants across the board used racial descriptors (e.g. ‘black’, ‘white’) when talking about the diversity of Colombians and linking diversity to regional variation; some also used the term ‘race’ itself. As we show in the next section, a clear contrast emerged here between LS people and HS people.

### Race, nation and the role of genetics in the constitution of collectivities

When it came to the relation between ancestry, genetics and race, the LS people tended explicitly to see race as related directly to genetic ancestry or genetics more broadly:

ESM: So would you say that genetic ancestry is the same as race?

Student 1: I would say so, yes.

Student 2: It all depends on the definition of race you have, but I would definitely say that genetics [i.e. a genome] is the mixture of many races [i.e. ancestries]. (FG-University C)

Yet most LS people also commonly elaborated on how regions configured people’s biology, culture and inclinations. Regional notions of difference organized the way bodily features, temperaments, dispositions and lineages were seen as linked to specific places inside the Colombian nation. In most cases, too, global geography implied race, and continents or regions of the world were immediately associated with a racial or colour group. Region was often linked to a specific genetic collective mark, as the following excerpt illustrates:

Student 1: Of course race and region coincide, because regions are different, if one region is hotter, then this will come out in the genes, genes will be of different colour …

Student 2: When you say black, Africa comes to my mind …

Student 3: Or when you say indigenous, Mexico, Bolivia or Peru come to my mind …

Student 1: I relate the white race with cold geographies, you know in the north and all those places …

ESM: So you think there is a relation between geography, race and genetics, right?

Student 4: Clearly there is a relation, if it was not the case there would be no heredity. Black parents could have a son with the phenotypical characteristics of a Chinese person. For me there is a clear relation, which is highly influenced by environmental and geographic factors that influence the appearance of a certain phenotype. (FG-University B)

Whiteness, blackness, indigeneity and admixture all have their corresponding original places inside Colombia, according to these informants. Typically the *paisas*^3^ from Medellín, Antioquia (in the north-west of the country), were seen as a good example of whiteness, while people from the Chocó province of the Pacific coastal region were commonly depicted as belonging to a Black lineage or population that might even be seen as relatively ‘pure’, despite the explicit disavowal of pure races. Indigeneity was located in the southern and Amazonian provinces of Colombia. Paisas and Chocoanos were seen as opposing poles in the national imaginary (in terms of racial configuration and social development, i.e. light-skinned Paisas were seen as more economically, socially and politically developed than Black Chocoanos).

HS people also recognized the regional diversity of the country and the links between, say, blackness and the Pacific coastal region, but they tended to resist talking about this diversity explicitly in terms of race and, more so, genetics; instead, diversity was expressed in an idiom of culture. In addition, they tended to see regional distinctions as stereotypes that categorized phenotypic diversity in ways linked to power and cultural hierarchies. For example,

If we talk about biology, we can only define the human race, the others are socially constructed to differentiate according to phenotype, and give more cultural attributes to some people, and generate inequality, assuming that the white race is superior and from there … the others are below, like in a pyramid. (FG-Afro-Colombian)

Still, even among the HS people, the common-sense links between regions and cultures were implicitly racialized, as – like most Colombians – they linked blackness, indigeneity and whiteness to specific certain regions of the country, even as they generally avoided the language of race and, especially, genetics. For both HS and LS people, race as purity was disavowed, but re-emerged via a link with regional difference in the nation. The difference was that the LS people made direct links between diversity, racialized genetics and region; the HS people linked diversity and region together, leaving the element of racialized genetics submerged.

The symbolic power of region was highlighted by the relative emptiness of the nation, seen as lacking unifying traits of Colombianness, which emerged from all our FGs. For the LS people, this was reflected in genetic as well as cultural terms: it was deemed possible to talk about the genetic composition of regional groups such as the Paisas, but it was nearly impossible to talk about the genetic composition of the average Colombian person:

Races on the Colombian coasts tend to be different from the people and races from the interior, we tend to see black races on the coast, and in the interior we are more pink- and white-looking and when you move to the south more types of indigenous races become apparent … within regions, people share a common background, heritage, customs and genetics, and I think that when it comes to Colombia, it is more a territorial, political division than anything else … a history in common, and some ideas of course, but that is all. (FG-University B)

I think Colombia does not have a particular genetic structure, I would say we share some customs and another few things but that is all … and maybe some cultural traits, but something like a characteristic Colombian genetics? No! (FG-University C)

For the LS people, even the possibility of purity itself re-emerged when we asked participants how they imagined genetic ancestry test results were produced by geneticists. These participants consistently answered that a ‘real’ or ‘pure’ African, European or Amerindian referent was used to define how much of each ancestry each individual had. These genetic referents were usually thought to be living in faraway places like Norway or sub-Saharan Africa, but many times relatively ‘pure races’ were thought to exist in indigenous, White or Black communities inside Colombia, said to be fundamentally a nation of mestizos. The Pacific coastal region, indigenous reservations and highland Antioquia were consistently seen in the LS FGs as places in which regionalism and a strong sense of belonging had kept genetic lineages relatively pure. Depicted as endogamous and closed off, these communities were seen as pure in comparison to ‘hot spots’ of recombination (mostly big cities). Bogotá was seen as having lost its sense of community and any endogamous mating practices (and thus a distinctive genetic profile). Many participants stated that ‘the most difficult thing to find in Bogotá are people who are originally from Bogotá’ (FG-University A).

In contrast, HS people challenged more systematically the idea of purity:

We think that when we talk of Africa, we talk of black people only, and that in Europe they are all white. But that is not the case: migrations and mixture have occurred there too. As it happens, in history those mixtures are erased; we should not forget that the *moros* [‘Moors’, or African-Arab settlers] lived for a thousand years in Europe. (FG-Afro-Colombian)

Nevertheless, in all our FGs, the idea that extensive *mestizaje* had undermined purity, and, as a consequence, race itself, was tempered by the continuous invocation of strong associations made between region and race. These links were made implicitly by the HS people and more explicitly by the LS people, who tended to invoke a genetic idiom as well.

## Race, nation and the role of genetics in the constitution of individuality

Despite the strong stereotypes that configure the Colombian nation(s) and the racialized populations inhabiting their so-called natural regions, when it came to the configuration of the individual the role of race and genetics was not fixed in a pre-determined way. In this section, we focus on the LS people and how they describe individuals as the product of fluid interactions between the environment and their genetic or ancestral baggage. For example, one of our participants stated,

Once I read at school that if the fourth generation of a person lives in a totally different environment from his ancestors, then that person would acquire some physical characteristics of the persons surrounding him. That is why we see that many black people who live in the United Kingdom are different from those living in Africa: for example their skin changes, they are not as black, not what you’d call really, really black anymore. (Biology student, FG-LATINA C)

She timidly added: ‘You might think I am crazy, but it seems to me that dark people become whiter as they become wealthier. … I can’t really explain how this happens but it kind of does’. For many LS participants, the individual could be understood at one level in terms of inter-connected aspects of race, genetics and appearance, but his/her individuality was constituted by multiple genetic, environmental, spiritual and socio-economic influences. Race was just one of many factors and, while at the regional level certain regular patterns were perceived to prevail, at an individual level things were much less predictable. It was believed that *mestizaje* in Colombia had been so thorough that almost any individual you picked would have a variety of Black, indigenous and White ancestors, a combination that introduced unpredictability.

Genetics and heredity were still deployed to explain particular experiences. A lot of participants talked about their spiritual and cultural affinities, and how they were rooted in a genetic background:

I think that likes and cultural traits are also hereditary, and I guess you could say genetic as well. … It has happened to me that you like something, and you don’t know why, and then you look and you find that someone else in the family liked that. (FG-University E)

Such ideas could also be powerfully racialized at an individual level. Blackness was believed to be a lot more persistent than whiteness or indigeneity, in terms of heredity, and if it was thought to exceed 5 percent or 10 percent of genetic ancestry it was deemed to be very visible in the features of the individual. On the contrary, indigeneity and whiteness were easily diluted, even if both were seen as more common among Colombians than Black or African ancestry. Latent traits, in the specific case of blackness, emerged, according to many of our informants, when they were exposed to what they considered to be iconic Black cultural products and practices: ‘When I hear the drums I feel like I am from over there: I guess that I have something black inside me’ (paraphrased from FG-University C and FG-Forensics A).

In order to organize the many possibilities opened by the all-encompassing idea of *mestizaje*, participants in our research resorted to three interpretative frameworks to define ancestry: somatic features, region and surname. The first and most common framework was a somatic reading of themselves and their families, which assigned certain parts of the body and face to racial groups. For example prominent bone cheeks were almost always related to having the look of an ‘*indiecito*’ (‘little Indian’); dark skin, a broad nose and a big or prominent behind (in both men and women) was almost always associated with being ‘Afro’ or ‘black’; and being ‘*mono*’ (fair-skinned) and ‘*ojiclaro*’ (green- or blue-eyed) was, not surprisingly, linked to being White or European. However, some somatic features, such as dark skin, occupied a more ambiguous space between ‘indigenous’ and the ‘black’ on the racial spectrum. Thus, there was plenty of room for debates about and interpretations of ancestral-somatic markers.

Most commonly, such ambiguities could be resolved by adding the two other interpretative frameworks – region and parentage/surname – to the equation (FG-University D):

Student A: I did it based on my last names, because I know the origin of my last names, therefore I gave myself 65% European ancestry, no indigenous ancestry, and 35% black ancestry.

Student B: Well, I consider myself to be 40% indigenous, 40% European and 20% black. I know that I don’t look black, but where I come from 60% of the population is black, and in my family my grandmother is not white. I don’t even know why I was born so white.

Student C: I can’t decide yet. I would be 75 indigenous, 25 European, because my dad is from Tolima, and that is a region with a lot of indigenous communities, but as it happens he has green eyes, and I met my grandmother from my dad’s side and she had lots of indigenous features. On my mother’s side my grandparents are white; white and blue-eyed. The story I have heard is that they came from Santander.^4^

Some informants counter-balanced what others might, by looking at them, deem to be their ancestral baggage by making reference to the physical characteristics of immediate ancestors. For example Student C used the bodily features of parents and grandparents to justify his ancestry, instead of reading it directly from his body. Some of those with medical and biological training used medical terms to justify ideas about their ancestry that seemed intuitively to contradict their somatic appearance. For example a medic told us: ‘Of course I have indigenous heritage, I have black eyes and my blood is type O+, something that is quite typical of indigenous people’ (FG-University A). This student’s accent and appearance led his Bogotá peers to nickname him *el paisita* (the little Paisa): the claiming of indigenous ancestry sent a clear message to his peers that he was not the ‘racist Paisa’ of Colombian regional stereotypes.

Somatic visible differences revealed the invisible (underlying) genetic reality of racial mixture, but not all genetic realities were expressed in the body. For example, someone who identified himself/herself with a certain region, family name and physiognomy as belonging to the category of ‘white-European’ would balance – or domesticate – this somatic reading with his/her own predilections for African rhythms or ideas about indigenous groups as the original people of Colombia:

I gave myself indigenous ancestry, because indigenous people are our common lineage, I identify with them, that is what is characteristic about Colombia, they are our roots, it’s a personal taste. (Police officer, FG-Forensics A)

I decided in part by affinities with some things. … The 20% of black I gave to myself, I did based on the time I spent living in the Caribbean coast, and I was much more sympathetic than what I initially thought to their food, their housing, the sea. So I said: this must be because of a black ancestor. (Forensic technician, FG-Forensics B)

These predilections and affinities revealed origins in a different non-somatic way, tied to personal experiences. However, some of our informants thought that genetics could finally prove or disprove these origins and believed that these inclinations were rooted in a genetic heritage. In the same sense, ascribing personal likes to genetic heritage was acceptable, or at least was not contested by other peers in the FGs.

Yes, because it has been seen that there are genes that modify the behaviour of a person, it has been seen that certain genes will give you your physical characteristics or [there are] genes involved in culture. (FG-University E)

It is just that DNA is everything. (Police officer, FG-Forensics A)

In sum, then, LS respondents, who easily linked race, place and genetics in quite mechanical ways when talking at a regional scale, deployed much more varied and flexible ideas about heredity when it came to explaining individual constitutions and identities, which brought them much closer to HS people. People brought different objects and elements of meaning – race, genetics, experience, place, predilection, parentage – into and out of proximity with each other, in flexible ways that submerged some elements, perhaps temporarily, while leaving a trace of their presence and the possibility of their reappearance.

### Domesticating genetic precision: Latency and manifestation

This flexibility at the individual scale of resolution could co-exist with a strong belief in the reliability of genetic knowledge, which was especially evident among the LS people, but was also present among others too, in terms of believing in the power of genetics to reveal hidden ancestry in the individual. (For example, despite scepticism and suspicion about the categories used in the ancestry test, all the Afro-Colombians we interviewed stated they would like to do a genetic test, mostly to uncover their lost African origins.) People with LS backgrounds were especially confident about the reliability of DNA:

RC (associate researcher): So what would make this blood sample different from other types of data about your origins or genealogy?

Participant: Because this is like a finger print, which is not going to be the same information that a person can give to you. It characterizes you in a special way. It gives you a register of what you really are! That means it is more definitive. I, as a biological engineer, know that there are genetic sequences that determine me, genetic markers that are used to do this type of research. It allows you to know characteristics that you cannot perceive *a simple vista* (just by looking). (FG-LATINA B)

The genetic tests carried out by the LATINA project scientists brought to the politics of belonging and identity calculable proportions of DNA, which could be seen as revealing a different and more ‘objective’ type of connection between history and a sense of communal and personal identity. This was illustrated during FGs when we asked about the differences between genetic science and other types of genealogical knowledge:

RC: So what makes the difference between genetics and, say, a genealogy?

Participant 1: It is the difference between the objective and the subjective. History alone can just explain to a certain level, there will always be limits. It [history] will never tell me, for example, you are from Achilles’ lineage. And genetics can. It provides much clearer percentages.

Participant 2: The historian is subjective, many persons can think many things, and everyone has their truth, and defends it, but the sample says ‘this is that, and behaves that way’; it is a tangible and logical manner to show what you really are. Although both can be complementary. (FG-LATINA A)

This faith in the reliability of genetic science was, however, tempered by concepts of latency and manifestation, according to which genes might express themselves or lie dormant. These ideas appealed to people with LS training, who could talk in terms of recessive and dominant alleles; but they also made sense to those from other backgrounds, who thought inherited traits could express themselves, perhaps unexpectedly, or remain latent – ‘I heard it is common for phenotypic traits to remain dormant, and then appear again in the third generation’ (FG-University H-2) (cf. Tyler, 2007). These concepts, when combined with the idea that *mestizaje* opens up an unpredictable range of genealogical possibilities, added to the indeterminacy around DNA at the individual scale of resolution.

Latency was used to explain counter-intuitive results. In many of our LS FGs when we asked a light-skinned person how they would react if they received an ancestry test that revealed that their ancestry was 90 percent African or Black, their faith in the reliability of DNA data sometimes led them to consider it a possibility (as long as the correct methods had been followed), even if unlikely. The question itself forced them to nuance a simple genetic determinism at the individual level and many LS people deployed genetic arguments about recessive genes and gene expression to explain the counter-intuitive:

I would think that maybe the little proportion that I have of European ancestry is determining my physical features, and the rest of my ancestry is determining genes that are not expressed phenotypically, that are not expressed in the colour of hair or in the colour of my eyes. I am going to go to the scientific field: practically about 90% of the genome is not expressed; the other 10% is functional. As I understand it, to determine ancestries you use SNPs [single nucleotide polymorphisms] that are part of non-codifying sequences of the genome, that is information that is going to be stored [in the body], and that you think will never become manifest, but equally it is going to mark your heredity, so these are genes that you have stored there, forgotten, but there. (FG-University C)

It is just that skin colour is affected by many factors: what I see is only a small reflection of what I have inside. In a way it is cultural, the black skin colour and broad nose are very suggestive of someone having black ancestry. But it is just that, suggestive: it does not mean that his/her genetic composition is always that. For example I have a brother with a very different skin colour, but I would not say we come from different places. Instead, I have to differentiate that one thing is what I am phenotypically, and another thing is what I am in my ancestry, where I come from. (FG-University B)

Irrespective of the economic or educational status of our participants, latency and manifestation were seen as the mechanisms, mostly framed as recessive or dominant genetic traits, that explained discrepancies between what we see with the naked eye and what is uncovered by the scientific gaze. Narratives of the ‘revelation’ of genetic truth and its ‘domestication’ by other factors were often framed in the idioms of genetic-phenotypic causation and genetic dormancy.

## Genetic ancestry and the politics of belonging and otherness

In general, HS people explicitly distanced themselves from interpretations that in any way explained identities and behaviours through genetics. For them, behaviours and dispositions were a question of political position and cultural differences – which could however entail the naturalization of inequality – and they were rarely seen as an innate characteristic of people or a result of biological factors.

However, a key pattern that emerged from the LATINA groups – and indeed many other FGs, including the humanities ones – was that people selectively and strategically used ideas about genetic ancestry to reinforce ideas and agendas that they already held, often in relation to person and family. New objects and categories – such as genetic *ancestría* – were incorporated into knowledge of family history and self. The indeterminacy of DNA at the individual scale of resolution allowed people to use it in very varied ways, which included both underwriting racial (and sometimes racist) determinism and challenging racist hierarchies.

Some of the participants in the LATINA project came to it in exploratory mode, to dig into their own *mestizaje*, to analyse and measure it: ‘I came here to know where I come from, to know what my origins are. I come from a very mixed family’ (FG-LATINA C). Others approached the project looking for something more specific in their family history. As one person said: ‘I want to know if I am a pure López’ (FG-LATINA B). Likewise, an amateur genealogist participant said: ‘this test will show and prove our genealogical connection with Captain Velázquez … a founding father of Antioquia, and our great, great, great grandfather’ (fieldnotes, 23 November 2011). For the latter two people, DNA could underwrite purity, rather than explain mixture.

A few thought genetics could help steer a future that was under threat of racial contamination or degeneration:

LATINA participant: I feel this can tell us all who we have been and where we are going.

ESM: How would you do that?

LATINA participant: You know, like if we have certain black, indigenous or white genes, and then we, at the personal and collective level, can think if we have the necessary things to progress. (Fieldnotes, 21 November 2011)

The implication here – rooted in long-standing racialized ideologies of *mestizaje* as a process of progressive whitening (Appelbaum et al., 2003) – was that ‘white genes’ would enable progress. This racialized genetic determinism was shared by some of the amateur Paisa genealogists we talked with, who thought people could use genetic ancestry data to make reproductive and social choices: ‘We know that indigenous people are astute, and many times dishonest and treacherous, [so] knowing the genetic component of some people could help you select who you should not adopt, befriend and marry’ (amateur genealogist, fieldnotes, 22 November 2011).

Many of our informants imagined that the test results could be used by some people in an assertion of racial superiority:

I guess all the Paisas that receive results with a high percentage of European ancestry will be delighted, probably displaying them in their windows, as they do with those announcements that declare theirs is a Catholic home, to boast and to promote their whiteness amongst the community. (Paraphrased from fieldnotes, FG-LATINA C and Introductory debate between LATINA organizers and High School Teacher, 19 November 2011)

In the context of Paisa politics of belonging, genetic ancestry testing, rather than being an enterprise to debunk racism, as many LATINA organizers hoped, was seen by many participants as a proof of origin very much aligned with popular ideas of Paisas as proud of their origins, ethnocentric and possibly racist.

These strategic uses of DNA were not confined to the LS people. Despite generally eschewing genetic determinism, some HS people deployed genetic knowledge for specific political purposes. A few people wanted to use genetic knowledge for subversive purposes: ‘I want LATINA to show them we all come from Africa, this test should be done to the many racists around, so they know they are also African’ (Afro-descendant high-school student, fieldnotes, 19 November 2011). In another case, genetics was invoked when a respondent felt that her appearance did not match her sense of identity. This White member of a group of Afro-Colombian activists in Bogotá went to great lengths to explain how her spiritual and cultural affinities made her feel deeply identified with her Black colleagues. She not only emphasized her political and social connections, which she considered the main reasons for her participation in the group, but also insisted that her affinity was in part borne of her knowledge of various African or Black ancestors in her family, and the fact that ‘we all come from Africa’ (FG-Afro-Colombian).

The hypothetical idea that a person might possess very high percentages of any ancestry was accepted even by many Paisas, who said they personally would not marry Black or indigenous people, but still considered that, whatever the genetic result thrown at them, DNA should be considered the bottom line and the truth about their ancestral origins. In these cases, the ‘truth’ about ancestry would not necessarily change their attitudes about mixed marriages: genetic evidence was being strategically side-lined, rather than deployed. A clear example of this was a LATINA participant – a self-declared White supremacist – who estimated that he had 100 percent European genetic ancestry. When asked, ‘What would you do if the genetic test came back reporting you have 15% European ancestry and the rest divided between black and indigenous ancestry?’, he responded,

I would ask for a second analysis … if it comes with the same percentages I will start researching if such thing is possible. There would be a great question – if I have those percentages, then why I am like this? And then, I would laugh at myself, burst into tears and I would think, this is not fucking possible … I would change my type of friends, because one is accepted in many places, because one is like this [indicating his blond hair and fair skin, but ignoring his dark eyes]. Normally if we find black people then we would beat them … imagine the change. … [After a moment of silence] I would have all these doubts, but I will still choose what I am in the moment, what I am, I will choose my beliefs, so I want to believe I am white, so be it – even though there is this contradiction. I would not choose the test … it might be the case that the numbers are scientific and objective, but for me my beliefs are more important than the numbers.

Although this man initially resisted the hypothetical scenario, he eventually said that he would simply ignore the scenario’s uncomfortable (to him) genetic data. If genetic knowledge contradicted preferred narratives of belonging, a process of avoidance and denial began, as in this case and also that of several amateur genealogists to whom we spoke.

## Conclusion

The dominant image of Colombia is that of a country in which a long history of *mestizaje*, which has produced a mainly mestizo population and culture, co-exists with a racialized geography of distinctive regions. This image was shared by all our FG participants, regardless of their background. However, the role genetic knowledge played in reinforcing this image and casting it into a genetic idiom turned out to be very uneven.

For some participants – located in the ‘certainty trough’ (Mackenzie, 1999) and who included students with some training in LS, forensic technicians, police investigators who were acquainted with forensic technologies and most participants in the LATINA genetic ancestry research project – the territories of genetics, belonging and kinship in Colombia were strongly coupled to the dominant imaginaries of racial and regional difference. These participants mostly rejected racism (although a few held openly racist views) and they also disavowed, in principle, the idea of pure races (although many referred to racial purity in practice). But, according to these participants, important and varied aspects of the nation and its human diversity, often understood in an idiom of racialized difference, were thought to be recorded in genetic structures. This coupling of belonging and genetics reinforces the dominant racial and national landscape, and reproduces the belief that genetic ancestry is a device that can reveal the more real, precise and generally hidden contours of race and nation. Genetic data entrained the possibility of unsettling this hegemonic landscape – by, for example, adducing the fact, much cited by LATINA researchers, that many mestizo Colombians are likely to have 95 to 99 percent indigenous ancestry in their mitochondrial DNA. But, while many of these participants could entertain the hypothesis that they might have high levels of African or indigenous ancestry, this did not seem to shift the basic contours of the dominant image of Colombia’s racialized regions. In this case, deference to scientific knowledge does not work out of ignorance, but thanks to some degree of familiarity with the technical principles underlying the science.

In contrast, HS people – humanities students and Afro-Colombian activists – treated genetics as a good deal less certain. Even though they might cede authority to genetics in terms of what it could reveal about individual ancestry, these people were more committed to alternative explanations of human variation, such as culture, which made the very categories used by genetic testing problematic. They resisted a racial idiom and profoundly challenged linking genetics to racial and regional difference at a collective level. For them, such links smacked of racism. Race had to be understood in terms of histories of power and oppression.

However, even among the FG participants who adopted relatively mechanistic views of the relation between genetics, race and region, notions of genetic determinations and of ‘truths’ revealed by genetic ancestry tests were domesticated by the idea that a person was constituted by a variety of processes in which genetic structures were not the only factor. This view was to be expected among the HS people, who tended to be suspicious of genetic determinism – especially when linked discursively to race – and to attribute more importance to culture in the constitution of the person. But among the LS people we also found an important difference in the way genetics was seen to shape human diversity when they came to ponder individual bodies and genealogical connections, as opposed to collective categories of region, race and nation. While DNA was seen as a material vessel that had the power to shape not only physical appearances but also emotions and spiritual inclinations, its power was mediated by many other factors.

Key here were ideas of dormant and expressive ancestries, which were used by many of our informants, often in the idiom of genetic science, to temper their mechanistic views. Latency and manifestation were used to account for multiple ways in which the individual was seen as fluid and not completely determined by genetics. Genetic dormancy and manifestation made it possible to re-interpret the ‘true’ racial-genetic source of each physical and psychological component. When the regional racial markers of belonging did not coincide with participants’ projects of self-fashioning, latency and manifestation allowed them to tap into the multiple possible connections of genetic ancestry and bring forth novel elements to define themselves. In this view, genetics was something like a set of components in a topological network, some of which become visible in our physical appearance or expressed in our likes and dislikes, while others remain submerged but may re-appear in different contexts. The view of genetic data as a malleable network of elements opened them up to strategic use – not only for the LS people, but also for the HS people. Despite their discomfort with genetic discourse, the latter groups might also use elements of genetic data to support certain agendas – such as the antiracist project of showing ‘racists’ that they too are Afro-descendants.

In attempting to understand the role played by the data emerging from recent genomic science and circulating through different public spheres, we need to be attentive not only to specific groups who have a vested interest in genetics – such as patient groups and other such focuses of biosociality (Heath et al., 2004; Rose, 2007) and roots-seekers (Nelson, 2008) – but also to a wider set of publics (Condit et al., 2004). In doing so, we need to be equally attentive to the variety among such publics in terms of their epistemological position: what kind of knowledge are they disposed to consider authoritative and in what ways? Our data show important differences in this respect between students who have been shaped by humanities and race-critical perspectives and those who come from other backgrounds, especially ones that involve some training in the LS. Although they may all agree that racism is undesirable, their underlying understandings of race and genetics are rather different. But, that said, we also need to pay attention to the specific domain in which people are thinking about issues of difference and belonging. Our data show that thinking these problems through at collective scales of resolution – the nation, the region, the community – tends to elicit higher degrees of racialized geneticization than when the same kinds of problems are pondered in relation to the person and his or her family genealogy.

## Notes

1. Only one focus group (FG) had two undergraduates and one postgraduate who were specializing in genetics.

2. All FGs were conducted by Ernesto Schwartz-Marín and Roosbelinda Cárdenas in the period between December 2011 and June 2012. All were audio-recorded, and included an informed consent process before they took place and a discussion afterwards.

3. *Paisa* is the term for people from the highland parts of the province of Antioquia; it denotes a regional group with a strong historical and cultural identity, linked to perceived whiteness and a history of economic dynamism, expansive colonization and entrepreneurial success.

4. Santander, like Antioquia, is a province linked by many of our research participants with whiteness and progress and specifically with the idea of law abiding citizens.

